# Watch me if you can: imagery ability moderates observational learning effectiveness

**DOI:** 10.3389/fnhum.2013.00522

**Published:** 2013-09-05

**Authors:** Gavin Lawrence, Nichola Callow, Ross Roberts

**Affiliations:** School of Sport Health and Exercise Sciences, Bangor UniversityBangor, Gwynedd, UK

**Keywords:** action observation, skill acquisition, motor learning, gymnastics, form tasks

## Abstract

Recent research has revealed similarities in brain activity during observational learning and motor execution. However, whilst action develops visual, motor and afferent representations during acquisition, action-observation has been proposed to only develop visual-spatial learning via visual representation. In addition, it has been suggested that the vividness of visual representations are determined by imagery ability. Thus, the purpose of the current investigation was to explore the possible moderating role of imagery ability in the effectiveness of observational learning. Participants (*n* = 40) were assessed on their imagery ability via the Vividness of Movement Imagery Questionnaire-2 (VMIQ-2) and then assigned to one of four groups; high imagery ability and observational learning (HIA-OL), low imagery ability and observational learning (LIA-OL), high imagery ability control (HIA-C) and low imagery ability control (LIA-C). Following group allocation all participants performed a pre-test consisting of five actual practice trials of a novel gymnastics routine. The HIA-OL and LIA-OL groups then participated in a 14 day observational learning intervention whilst the HIA-C and LIA-C groups acted as controls. Following this, participants performed a post test, which was identical in nature to the pre-test, before finally completing the VMIQ-2 again. Performance on both the pre-test and post test was evaluated by two qualified gymnastics judges. Results revealed that gymnastics performance increased from pre-test to post test for both the HIA-OL and LIA-OL groups. However, this effect was greater in the HIA-OL group suggesting that the relationship between observational learning and successful imitation performance is moderated by imagery ability.

## Introduction

Following recent improvements in the measurement (e.g., PET, fMRI) of cognitive activity in the brain researchers have begun to study the neural correlates between both action observation and movement imitation (Decety et al., 1997; Grezes and Decety, 2001; Filimon et al., 2007). Research in this area has revealed the activation of common neural structures (e.g., Parieto-frontal areas, cerebellum and supplementary motor area (SMA)) between observational learning (OL) and physical practice in both healthy (Macuga and Frey, 2012; Nedelko et al., 2012; Szameitat et al., 2012) and patient populations (Szameitat et al., 2012). Furthermore, recent neuroimaging (Macuga and Frey, 2012) and behavioral research (Ong and Hodges, 2010; Boutin et al., 2012; Ellenbuerger et al., 2012; Hayes et al., 2012a,b) suggests that the activation of these brain regions serve different purposes during OL and actual practice: actual practice results in the development and coding of visual, motor and afferent neural representations of the to-be-learned task, whereas OL leads to the encoding of visual representations (Boutin et al., 2012; Ellenbuerger et al., 2012; Hayes et al., 2012a,b).

The rationale for the development of different neural representations between OL and movement execution derives from proposals that the sensory and motor processes involved in these two paradigms operate differently. That is, learning through movement execution involves the understanding, analyzed and adaption of the interacting effects between the efferent neural commands and afferent neural information (for a review see, Elliott et al., 2010). For example, in order to accurately acquire executed motor skills individuals need to develop internal feed-forward and inverse models. These compare the expected and actual feedback to map the correct movement commands to the required sensory consequences and transform sensory consequences into the correct motor commands, respectively. Whilst actual practice involves all of these motor and sensory transformations, OL affords only some since the observer experiences the same visual input as those in movement execution but does not experience the processes involved either in sending neural commands to the motor periphery or in receiving resultant afferent feedback from movement.

In partial support of the above, Voisin et al. (2011) revealed that OL does not result in actual muscle contraction as measured by EMG. However, EEG data revealed that OL of either a hand action or a hand being passively touched by an object resulted in a modulation of somatosensory activity. Given the lack of EMG activity during OL, the somatosensory modulation most likely resides in processes involved in internal feed-forward models of control. That is, because no actual afferent information is present in OL modulation of activity in somatosensory areas cannot occur due to processes involved in inverse models of control. Rather, the subliminal activation of brain regions during OL may result in efferent copy development and thus development of feed-forward models of control. In support of the possibility that OL enhances feed-forward models, research has revealed that corticospinal activation (measured via motor-evoked potentials; MEP) is greater during OL compared to at rest (Brighina et al., 2000; Aziz-Zadeh et al., 2002; Clark et al., 2004) with the temporal structure of these MEPs sharing the same structure as the muscle phases involved in actual physical practice (Gangitano et al., 2001).

Whilst, OL may well invoke development of the efferent processes involved in motor representations, recent empirical evidence suggest that the sensorimotor processes underpinning OL involve visual rather than complete (i.e., both feed-forward/efferent and inverse/afferent) motor representations (Hayes et al., 2012a,b). Hayes et al. investigated the processes subserving both OL and learning that involves motor execution using both intermanual (Hayes et al., 2012a) and intramanual (Hayes et al., 2012b) transfer paradigms. In the intermanual paradigm, participants learned both the absolute and relative timing of a movement sequence with their right arm either through motor execution or observation before being instructed to perform the same absolute and relative timings with their left arm (i.e., intermanual transfer). Whilst both groups learned the absolute and relative timing of the task equally well when asked to reproduce the same visual-spatial pattern, performance in transfer was significantly lower in the observation group. The intermanual transfer involved the production of a mirror image equivalent to that learned with the right limb. Thus, the authors concluded that the superior performance of the motor execution group was due to this mirrored image engaging homologous muscles to those involved in practice and the motor representations developed therein, whereas, the lower performance of the OL group indicated the development of a visual-spatial (rather than a motor) representation was ineffective in the mirrored task (transfer). To corroborate these suggestions the authors replicated their previous experiment but included an intra- rather than intermanual transfer paradigm (i.e., the transfer task required use of the same limb to that involved in learning, but modification of the scaling between the movement of the limb and the visual-spatial outcome). The rationale being that transfer performance would now be superior in the OL group because the visual-spatial representation formed during practice would be congruent with that required during transfer. Whereas, performance of the motor execution group would be attenuated because the motor representation developed during practice would need to be adjusted to meet the novel motor execution and sensory consequences of the transfer test. Similar to the intermanual experiment, results revealed that both the motor execution and OL groups demonstrated equivalent retention performance. However, participants in the OL group were better able to adapt to the modifications in gain between their limb movements and the associated visual consequences in the transfer test supporting the proposal that learning through action observation results in the development of visual-spatial task representations.

The process of manipulating these types of mental representations (i.e., visuo-spatial images) has been conceptualized in terms of forming, transforming, and maintaining the image (Kosslyn, 1994). These processes not only support the learning and execution of motor performance (e.g., Hardy and Callow, 1999; Fourkas et al., 2006; Guillot et al., 2008, 2009), but also many other important aspects of human functioning. For example, imagery is implicated within working memory (Bywaters et al., 2004), problem solving (Hegarty and Kozhevnikov, 1999) creative thinking (LeBoutillier and Marks, 2003) and language (Bergena et al., 2007). However across these areas, individual differences in imagery ability (e.g., vividness) influence the effectiveness of imagery on functioning (e.g., Gonzalez et al., 1997; Baddeley and Andrade, 2000). Indeed, behavioral research has demonstrated a moderating effect of vividness on motor learning and execution (e.g., Goss et al., 1986; Robin et al., 2007) such that individuals with better imagery ability receive more benefit from imagery use.

Although recent neuroimaging studies have shown differential neural activity and concomitant increased motor output and performance related to ability of imagery (Cui et al., 2007; Guillot et al., 2008; Logie et al., 2011; Williams et al., 2013), a mechanism by which the neural differences underpinning vividness may cause these differential behavioral effects has not been offered. However, a cognitive rationale can be provided. Specifically, a more vivid image provides the imager with clearer information regarding what she or he has to execute via a more detailed visual-spatial representation in working memory (Baddeley and Andrade, 2000), with research indicating that the more detailed the visual-spatial representation, the greater the behavioral response (Callow et al., 2006).

As OL enhances learning and skill development through the development of visual-spatial representations (Hayes et al., 2012a,b) and that ones’ ability to produce a vivid image impacts the quality visual representation in working-memory (Baddeley and Andrade, 2000) one might expect that the ability to image (i.e., to create vivid and realistic visual images) might moderate the effectiveness of OL. However, to the best of our knowledge, this proposal has yet to be tested in the literature. Thus the primary purpose of the present study was to investigate whether imagery ability moderates the OL-performance relationship. Further, as OL increases how vivid an image is (Rymal and Ste-Marie, 2009) and also the ease of imaging a movement (Williams et al., 2013), the secondary purpose of the study was to further explore the effect that observational learning has on imagery ability. To examine these aims, participants learned a gymnastic floor routine via an observational learning paradigm and completed a widely used measure of imagery ability Vividness of Movement Imagery Questionnaire-2 (VMIQ-2; Roberts et al., 2008) before and on completion of the intervention. We hypothesized that those participants with higher imagery ability would achieve greater learning, as measured by retention, compared to those participants with lower imagery ability, and that OL would increase imagery ability.

## Materials and methods

### Participants

Eighty four participants (*n* = 43 males and 41 females) aged between 18 and 26 (M = 19.8; SD 1.3) volunteered to participate in this experiment. All participants self-reported no previous experience in gymnastics, were naïve to the research hypotheses being tested and gave their consent prior to taking part in the investigation. The experiment was conducted in accordance with the institutions ethical guidelines for research involving human participants. Since the investigation required a high and low imagery ability sample population and due to the experimental task being form based (e.g., a gymnastics routine), participants were screened in regards to their imagery ability and preference. This was achieved via completion of Callow and Roberts (2010) revised VMIQ-2 (see below for specifics). Only those participants with either a high (VMIQ-2 score < 26) or low imagery ability (VMIQ-2 score > 36) and a preference for external visual imagery (EVI) were selected.^1^ Following this procedure the investigation was left with 40 participants (12 males, 28 females) aged between 18 and 26 (M = 20.1; SD 1.7) with an equal number of high and low imagery ability participants.

### Task and apparatus

In order to measure imagery ability, participants completed the revised VMIQ-2 (Callow and Roberts, 2010). The revised VMIQ-2 requires athletes to form images of a variety of movements and then rate the vividness of each image. Specifically, the measure contains 12 items and participants are asked to image each item from a specific imagery perspective and rate the image on a 5 point Likert scale according to the degree of clearness and vividness (from 1; perfectly clear to 5; no image at all). The 12 items are then added together to give a score for that imagery subscale. A lower score indicates greater imagery ability. This process is completed separately for External Visual imagery, Internal Visual imagery and Kinesthetic imagery. The questionnaire also requires participants to use a 1–10 (1 = strong internal preference, 5 = no preference, and 10 = strong external preference) Likert scale to rate the extent to which they have a preference for a particular imagery perspective. For the purposes of the current experiment only those individuals who reported 7 or above (≥ moderate external preference) on this question were selected for participation. The VMIQ-2 displays acceptable factorial, concurrent and construct validity (see Roberts et al., 2008).

**Figure 1:**
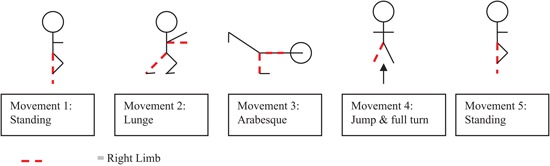
**Schematic representation of the five separate gymnastic movements contained within the gymnastics floor routine**.

The experiment took place in a gymnastics hall in which two standard multipurpose gymnastics mats (2m × 1m × 50 mm) were set out horizontally. Marker tape on the mats was used to identify the start position and movements were recorded on a Sony Digital Video Camera Recorder (DCR-DVD106) mounted onto a tripod located at a distance 3.5 meters away from participant and at an angle of 45° (0° was taken as the center of the participants navel). At the start of the experiment, participants were shown a short video ten times^2^ on a television monitor (Aiwa VX-G142) of an expert gymnast performing a floor routine. The perspective of the expert in the video was consistent to a third person view or external perspective. Participants were instructed that performance was being measured by how accurately they were able to reproduce the movement form within the video. The routine (see Figure 1) consisted of five simplistic movement components each of a comparable level of difficulty, as listed by the *Fédération Internationale de Gymnastique* (2009).^3^ Specifically, the movements consisted of a starting position, a lunge, an arabesque, a full turn and a finish position, which were all held for three seconds. For the start position participants were required to balance on their right foot with their left leg bent and their left foot resting on their right knee. Participants had to hold their arms out horizontal in front of their body with their left arm at 45° and their right arm out in front. They had to hold their hands with their palms facing down and their fingers straight. For the lunge participants were required to step forward onto their left foot holding their right leg back straight with their body upright; arms horizontal in front of the body and palms facing down. For the arabesque participants were required to stand on their left leg, with their right leg behind, horizontal and straight, and foot pointed. They then had to circle their right arm back until straight behind the body, while holding the left arm horizontal and straight in front of the body, before returning to standing position. For the full turn participants were required to jump in the air swinging their arms forward and overhead for momentum. Participants had to turn their head in the direction of rotation (right), pulling with the opposite shoulder and hips to execute a 360° turn in the air, before landing on two feet, with their arms horizontal in front and palms facing down. The finish position was identical to the starting position.

### Procedure

Participants were placed into one of two categories as defined by their score on the VMIQ-2; high imagery ability (VMIQ-2 score < 26; 7 males, 13 females) and low imagery ability (VMIQ-2 score > 36; 5 males, 15 females). Participants within these categories were then randomly assigned to one of two further subcategories. This process resulted in four experimental groups; high imagery ability and observational learning (HIA-OL; 4 males, 6 females), low imagery ability and observational learning (LIA-OL; 3 males, 7 females), high imagery ability control (HIA-C; 3 males, 7 females) and low imagery ability control (LIA-C; 2 males, 8 females). Following group allocation all participants watched the video of the gymnastic routine and immediately performed a pre-test that consisted of one block of five trials of the gymnastics task.

Participants in the HIA-OL and LIA-OL groups then participated in a 14 day observational learning intervention whilst the HIA-C and LIA-C groups acted as controls. Specifically, both the HIA-OL and LIA-OL groups were required to return to the gymnastics hall every day for a period of two weeks in order partake in the observational learning intervention. This consisted of watching the video clip of the expert gymnast 20 times on each visit with a 30 second period between each clip presentation (participants in the HIA-C and LIA-C groups did not receive an intervention). Following this 14 day phase, all participants were given a period of 1 day before performing a post test which was identical in nature to that of the pre-test. Finally, participants were asked to complete the VMIQ-2 for a second time.

### Dependent measures and analyses

All trials were video recorded for analysis. Performance was assessed independently by two experienced gymnastics judges (British Gymnastic Association (BGA) area qualified (Welsh Gymnastics) with 22 years experience and BGA club qualified with 10 years experience, respectively) who were blind to both the research hypotheses and experimental groups, and were not present during testing. Participants were judged according to the *Fédération Internationale de Gymnastique* Code of Points (2009) for Women/Men Artistic Gymnastics (WAG/MAG). Judges were asked to view the video recordings and award points for each trial according to the criteria on the** Code of Points, with marks deducted for poor execution and errors. A maximum score of 10 points could be awarded for the whole routine (this was a composite score for all five movements). In order to assess reliability of judging, mean inter-judge reliability scores were calculated and analyzed across all trials. The results of this analysis revealed a significant correlation (*r* = 0.901, *p* < .001), suggesting that participants’ performance was rated similarly across both judges for each trial. Following this analysis, the mean of the two independent judges scores were calculated for each trial for each participant. These data were then used to calculate a single mean for the 5 pre-test trials and a single mean for the 5 post-test trials.

To ensure there were no significant differences between the performances of the groups prior to testing, the means of pre-test performance data were submitted to a 4 group (HIA-OL, LIA-OL, HIA-C, LIA-C) one way ANOVA. In order to assess the gymnastics performance data and the imagery ability data VMIQ-2 separate 4 group (HIA-OL, LIA-OL, HIA-C, LIA-C) × 2 experimental phase (pre-test, post-test) ANOVAs with repeated measures on the second factor were conducted. Significant between-subject effects were broken down using Tukeys HSD post hoc tests (*p* < .05) while significant within-subject effects were broken down into their simple main effects (*p* < .05).

## Results

### Pre-test

The one-way ANOVA conducted on the pre-test gymnastics performance data revealed a non significant between group difference, *F*_(3, 36)_ = .53, *p* = .67 (HIA-OL mean = 3.16; HIA-C mean = 2.56; LIA-OL mean = 3.10; LIA-C mean = 2.52). Thus any performance differences at postest cannot be attributed to undue variances between the groups.

### Gymnastics performance

**Figure 2:**
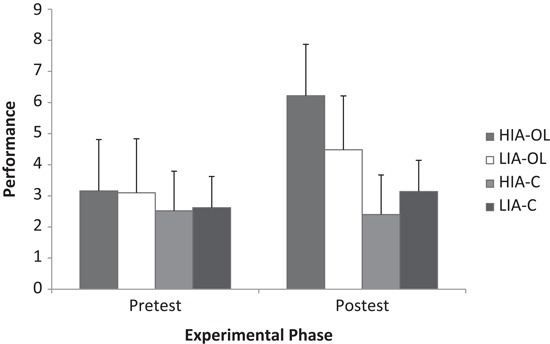
**Gymnastics performance as a function of group (HIA-OL = high imagery ability observational learning; LIA-OL = low imagery ability observational learning; HIA-C = high imagery ability control; LIA-C = low imagery ability control) and experimental phase**.

The analysis of the gymnastics performance data from pre-test to post-test reported significant main effects for experimental phase (*F*_(1, 36)_ = 174.66, *p* < .001, *η_p_*^2^ = .83) and group (*F*_(3, 36)_ = 4.99, *p* < .01, *η_p_*^2^ = .29), together with a significant experimental phase × group interaction (*F*_(3, 36)_ = 56.62, *p* < .001, *η_p_*^2^ = .83). Breakdown of the interaction revealed that only those groups that had experienced the observational learning intervention increased their performance pre to post test (HIA-OL *t*(9) = −11.06, *p* < .001, *i* − *j* = 3.06, *d* = 1.83, *r* = .67; LIA-OL *t*(9) = −6.39, *p* < .001, *i* − *j* = 1.38, *d* = 0.82, *r* = .38), with this increase being significantly greater in the high compared to the low imagery ability group (see Figure 2). Specifically, whilst performance at pre-test did not significantly differ between groups, performance at post-test was significantly greater in the HIA-OL and LIA-OL groups compared to the control groups, with performance in the HIA-OL group (mean = 6.22, SD ± 1.71) being significantly greater than that of the LIA-OL group (mean = 4.48, SD ± 1.65) (*p* < .05).

In order to corroborate the breakdown of the experimental phase × group interaction, the pre to post test change score was calculated for each group and submitted to a one way ANOVA. Results revealed a significant difference between the means (*F*_(3, 39)_ = 56.62, *p* < .001), with Tukeys HSD indicating significantly greater change scores in the HIA-OL group compared to LIA-OL, HIA-C, and LIA-C groups (*i* − *j* = 1.68, *p* = < .001, *d* = 2.15, *r* = .73; *i* − *j* = 2.54, *p* = < .001, *d* = 3.94, *r* = .89: *i* − *j* = 3.18, *p* = < .001, *d* = 5.05, *r* = .93, respectively). Furthermore, the change scores in the LIA-OL group were significantly greater than those of both the HIA-C *i* − *j* = .86, *p* = .011, *d* = 1.66, *r* = .64) and LIA-C (*i* − *j* = 1.5, *p* = < .001, *d* = 3.00, *r* = .83) groups. The control groups were not significantly different to one another (*i* − *j* = 0.64, *p* = .082).

### Imagery ability (VMIQ-2)

Analysis of the VMIQ-2 data from pre to post test did reveal a significant main effect of experimental phase (*F*_(1, 36)_ = 17.12, *p* < .001, *η_p_*^2^ = .32), and group (*F*_(3, 36)_ = 154.13, *p* < .001, *η_p_*^2^ = .93), with scores being significantly lower (indicative of better imagery) post test (pre-test mean = 27.90, post test mean = 25.90) and, not surprisingly, in the high compared to low imagery ability groups. No interaction between the two factors (*F*_(3, 36)_ = 2.20, *p* = .11, *η_p_*^2^ = .16) was observed.

## Discussion

The present study examined the moderating role of imagery ability on the relationship between OL and performance. Because the effects of OL on motor execution are a function of the visuo-spatial representations developed during learning (Boutin et al., 2012; Ellenbuerger et al., 2012; Hayes et al., 2012a,b) and that the ability to produce vivid and realistic images impacts the quality of visual representation in working-memory (Baddeley and Andrade, 2000), we expected OL to be more beneficial to learning for individuals with high, as opposed to low, imagery ability.

Results revealed that the benefits of OL were significantly greater for participants with higher levels of imagery ability. Specifically, whilst only those groups that had experienced the observational learning intervention increased performance from pre test to post test, this increase was significantly greater in the high compared to the low imagery ability group. These findings indicate that the effectiveness of OL is indeed moderated by the ability to produce a vivid image. Hayes et al. (2012a,b) revealed that the absence of sensorimotor reafference during action-observation enables actions to be represented in visual spatial coordinates only. That is, because participants are at rest during OL they are not directly afforded afferent information from which to develop inverse models (i.e., information involved in the planning and updating of future motor commands). However, despite these OL motor representation limitations, the retention performance (i.e., the exact repetition of the same observed action) following an OL intervention is often similar to that of actual practice (Boutin et al., 2012; Hayes et al., 2012a,b). These results indicate that the direct visuo-spatial replication of the observed movement pattern is possible regardless of whether actual or observed practice interventions have previously been followed. The performance findings of the present investigation suggest that the visuo-spatial replications of the task are more effectively developed in individuals with higher imagery ability. Since visuo-spatial task replications are suggested to be utilized for feed-forward control (Hikosaka et al., 1999) it appears that action-observation developed feed-forward models of motor control are moderated by one’s ability to produce vivid images of imagined actions. The same may also be true for the enhanced somatosensory (Voisin et al., 2011) together with corticospinal (Brighina et al., 2000; Aziz-Zadeh et al., 2002; Clark et al., 2004) activity in the absence of actual motor activation during OL. That is, the efferent copy development as a result of subliminal activation of brain regions during OL (Voisin et al., 2011) may be moderated by the imagery ability of the participant. Indeed, if OL is due to somatosensory representation or corticospinal activity alone, then one would not expect to observe greater benefits of OL for individuals with high compared to low imagery ability. As such is it likely that OL involves the development of processes involved in efferent copy and visuo-spatial representation.

Hikosaka et al. (1999) propose that the acquisition of movement patterns involves two distinct, simultaneously developing phases of learning; a fast developing cognitive phase where movements are coded in visual-spatial representations and a slower developing phase where movements are coded in motor representations. Since the OL benefits of the present investigation were moderated by imagery ability, it is possible that the participants with higher imagery ability were able to develop these cognitive visual-spatial representations at a faster rate than their low imagery ability counterparts. In support of this proposal, research has indicated that the SMA, an area that has a dense population of visual coding cells (Georgopoulos, 1991) and plays a critical role in coding a motor response based on visual information (Hayes et al., 2012b), demonstrates greater cortical activation in OL and imagery interventions compared to OL interventions alone (Macuga and Frey, 2012; Nedelko et al., 2012). As such, the increased activation in the SMA associated with imagery may result in more effective and/or faster coding of visual-spatial representations allowing participants with high imagery ability to acquire the cognitive phase of Hikosaka et al. (1999) model at a quicker rate than participants with low imagery ability.

Whilst the current research adopted a 14 day OL intervention, the total duration of the actual observation within this intervention was approximately 140 min (i.e., 20 observations of ~ a 30 second video per day). Since the present investigation suggests that a possible mechanism for the moderating role of imagery ability on the benefits of this intervention resides in participants developing faster cognitive visual-spatial representations, future research should consider investigating whether longer OL interventions would see a gradual reduction in the moderating role of imagery ability. This is in line with the proposal that the slower developing motor code representation in Hikosaka et al. (1999) model dominants over the visual-spatial representation later in practice. In addition, research has suggested that the amount of practice is thought to be a critical factor to determining when a performer will move from a visuo-spatial to a motor representation for learning (Park and Shea, 2005; Kovacs et al., 2009). Thus, when participants with high imagery ability have completed enough practice to reach a stage where the motor representation phase dominates, it is reasonable to conclude that the benefits of the faster developed (in comparison to those with low imagery ability) visual-spatial representation would be reduced. However, that is not to say that the benefits of OL would be removed following extensive levels of practice, indeed research has revealed the opposite (Stefan et al., 2005; Ray et al., 2013), but rather that the benefits associated with imagery ability would be reduced.

Recently, Williams and Cumming (2012) have also revealed links between OL and imagery. Specifically, the researchers suggest that individuals with high levels of imagery ability demonstrate greater use of both imagery and OL compared to their low imagery ability counterparts. Because the current investigation did not adopt any manipulation checks it is possible that the greater performance at post test of the HIA-OL compared to the LIA-OL group is due in part to participants in the HIA-OL group utilizing imagery during the 14 day OL intervention period. Since it is widely accepted that the use of imagery enhances performance (for a review see, Cumming et al., 2008), this strategy would likely lead to increases in post-test performance. A second potential limitation within the current experimental design is associated with a possible attention effect within the control groups. That is, participants in the HIA-C and LIA-C groups were not required to visit the laboratory during the 14 day OL intervention. Although, all participants were not explicitly aware of the number of groups or the different treatments that the groups received, it is possible that the choice not to include a placebo intervention for the control groups may have resulted in an amotivating effect and a reduction in post-test performance compared to the OL intervention groups.

As well as performance effects, our data demonstrated significant improvements in imagery ability as a result of the OL intervention. While not the primary purpose of the study, these findings do corroborate previous work (see Rymal and Ste-Marie, 2009). Given that imagery ability moderates the effectiveness of imagery on human functioning, as well as OL, ensuring that individuals intending to use these particular cognitive strategies are able to image to a reasonable degree is paramount. Indeed, recent work (e.g., Williams et al., 2013) has demonstrated how imagery training programs can increase imagery ability, and the present investigation provides another useful approach to enhancing this important ability. Due to their apparent simplicity (i.e., watching a demonstration/model) it may be that OL interventions are particularly useful for developing the imagery ability of individuals who have very poor imagery ability or for various clinical populations (e.g., stroke), although this suggestion is somewhat speculative.

To conclude, the present study demonstrates that imagery ability moderates the effectiveness of OL on the acquisition of a motor sequence. The mechanism by which this benefit occurs is likely due to increases in the activation of brain regions (e.g., SMA) associated with the development of visuo-spatial representations deemed particularly important for movement pattern acquisition early in learning (Kosslyn, 1994). This moderating role of imagery ability on OL effectiveness is a novel finding, as such future research should aim to collaborate these effects together with explicitly elucidating the underlying mechanisms involved in order to further advance our understanding of when and how OL is most effective.

## Conflict of interest statement

The authors declare that the research was conducted in the absence of any commercial or financial relationships that could be construed as a potential conflict of interest.

## Footnotes

^1^The rationale here was that EVI is the most efficacious form of imagery for form based tasks (Hardy and Callow, 1999).

^2^This frequency of demonstration was in accordance with Weeks and Choi (1992) who suggest that this number has been shown to be sufficient for the performer to create an accurate depiction of the skill in their mind (also see, Lawrence et al., 2011).

^3^Fédération Internationale de Gymnastique: The code of points for acrobatic gymnastics 2009-2012. Fédération Internationale de Gymnastique, Suisse. http://www.fig-gymnastics.org
